# A case report: Pavlovian conditioning as a risk factor of heroin 'overdose' death

**DOI:** 10.1186/1477-7517-2-11

**Published:** 2005-07-25

**Authors:** József Gerevich, Erika Bácskai, Lajos Farkas, Zoltán Danics

**Affiliations:** 1 Addiction Research Institute, Budapest; 2 ELTE University, Faculty of Orthopedagogics, Budapest, Hungary; 3 National Institute of Psychiatry, Budapest, Hungary

## Abstract

**Background:**

The authors present a case illustrating a mechanism leading directly to death which is not rare but has received little attention.

**Case presentation:**

The case was evaluated by autopsy, investigation of morphine concentration in the blood, and clinical data. The heroin dose causing the 'overdose' death of a young man who had previously been treated a number of times for heroin addiction did not differ from his dose of the previous day taken in the accustomed circumstances. The accustomed dose taken in a strange environment caused fatal complications because the conditioned tolerance failed to operate. The concentration of morphine in the blood did not exceed the level measured during earlier treatment.

**Conclusion:**

These results are in line with the data in the literature indicating that morphine concentrations measured in cases of drug-related death do not differ substantially from those measured in cases where the outcome is not fatal. A knowledge of the conditioning mechanism can contribute to prevention of fatal cases of a similar type. The harm reduction approach places great stress on preventive intervention based on data related to drug-related death.

## Background

A number of mechanisms leading directly to drug-related death are known. One of the most widely known variants is where the active substance content of a drug bought on the black market differs from the accustomed level [1]. Lethal development related to drug overdose occurs most frequently when the patient accustomed to the drug gives up its use then after a while attempts to continue addictive behaviour with the same dose used immediately before withdrawal [2]. The use of drugs in combination also increases the danger of a fatal overdose [3].

However, there is also another explanatory model of cases of drug-related death. Siegel et al. showed that situation-specific tolerance is capable of preventing the fatal consequence of a fatal-sized opiate overdose. When rats are given a large dose of morphine following morphine dosing in an environment substantially differing from the one in which they experienced the effects related to morphine, signs of overdose rapidly appear and in a few cases lead to the death of the rat. In contrast, in the case of rats where the morphine is dosed in the same circumstances the same size dose has a substantially smaller effect since the substance was given in the accustomed environment and so they were "expecting" its effect [4].

Siegel interviewed 10 heroin overdose survivors in an attempt to ascertain whether the overdoses occurred following novel pre-drug cues. For seven of the overdoses, the drug was administered in an environment not previously associated with drug use [5].

O'Brien showed the conditioned tolerance phenomenon in detoxicated heroin addicts in a double blind situation, on four different occasions. On one occasion the subjects were given a moderate dose (4 mg) of hydromorphon in an infusion without knowing what they were being given and when. On the second occasion they injected the same dose themselves. On the second two occasions the same process was repeated with salt. When they were given the opiate without prior indication, the subjects showed a significantly greater physiological reaction following the full effect of the drug than when they knew what they were receiving (since they injected it themselves). The anticipation and preparation for taking the drug triggers responses contrary to the drug effect in persons already showing drug tolerance. The anticipation preceding the administration of opiate, acting as a conditioned stimulus, reduced the action of the drug and so contributed to the development of a mechanism corresponding to tolerance [6].

Gutiérrez-Cebollada et al. interviewed 76 heroin addicts admitted to the emergency room of a university hospital in Barcelona. Fifty-four patients were admitted because of heroin overdose, and 22 were seeking urgent medical care for unrelated conditions, but their interview revealed intravenous heroin self-administration 1 hr or less before admission. All of the patients who had recently used heroin, but had not suffered an overdose, injected the drug in their usual drug-administration environment. In contrast, 52% of the overdose victims administered "in an unusual setting" [7].

The case described here is the first in the literature of addiction medicine where death can be quite clearly attributed to Pavlovian conditioning.

## Case presentation

K.J., a 26-year-old male, first presented at the Drug Prevention and Treatment Centre with his wife in November 1997. They both asked to be treated for heroin addiction. Before admission they had been treated once as out-patients without success. He first used heroin a year later, in 1995, intravenously from the start, beginning with half a gram once a week; six months later his dose had increased to a gram a day. By then he was shooting up daily. He had never had any physical illness. Once he was hospitalized because of overdose, although opiate antagonist medication was not necessary. The concentration in the blood of morphine, the catabolite of heroin, was 0.05 mg/l. At the time of admission no internal medicine or neurological disorder could be found, while dysthymia and emotional lability were observed in the psychiatric state without psychotic symptoms or disorientation. Laboratory tests showed no abnormality. Detoxification with clonidine was followed by rapid relapse. He was never abstinent for longer than a week.

His wife recounted that on January 8, 1999, the day before his death, they had decided to begin withdrawal the following day. Next day, January 9, the wife remained at home and K.J. set out for work. What happened after that can be reconstructed from the forensic medical report and from information given by drug-using friends. On the way to work K.J. changed his mind and, breaking his promise to his wife, went to the dealer and bought a dose of heroin. He met other drug-using friends there who had bought heroin from the same dealer that day and later told the author that the heroin purchased then did not differ in quality from the usual. K.J. did not return home with the heroin purchased as he did on other occasions but went to the public toilet in the pedestrian underpass at the Népliget Metro station where he injected the same quantity (0.5 gram) that he had taken the previous day in the accustomed place, at home with his wife. The authorities called out were unable to help and pronounced him dead. A syringe half filled with a yellowish-brown fluid and a sooty spoon were found beside the body. The fluid in the syringe was heroin, while the metabolite of heroin, 6-0-acetylmorphine, and morphine-3-0-glucuronid were found in the blood and urine.

The autopsy found numerous traces of punctures by injection needles of various age on both upper limbs, the left side of the neck and the lower limbs. Traces of an infected but healing needle puncture were found inside the right elbow. Examination of the internal organs showed signs of general, very acute circulatory failure: acute congestive plethora of the organs, cerebral oedema, heightened brain pressure, cerebellar inclusion, acutely inflated lungs. The concentration in the blood of morphine, the catabolite of heroin, was 0.05 mg/l. The dose did not differ from the accustomed, daily dose. Other substances (alcohol, benzodiazepines, barbiturates) were not found. Heroin 'overdose' was given as the cause of death.

## Conclusion

The fatal consequence of the heroin injection may have been caused by the failure in the action of conditioned tolerance. As the figure shows, when a conditioned place preference arises, the user has to take a bigger dose each time to achieve the same effect as the user who does not have the opportunity for secondary conditioning with environmental stimuli since he or she constantly changes the place where the drug is taken [6]. When the drug is taken in a strange environment the conditioned tolerance does not operate since the organism is not "expecting" the drug. The end result is that the otherwise accustomed dose leads to an overdose and thereby to death. This is why the term "overdose" is misleading since the quantity taken was not greater than other doses taken without fatal complications [8].

**Figure 1:**
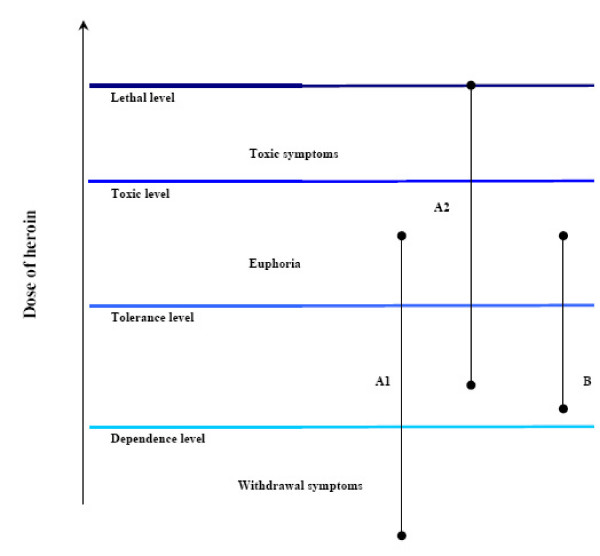
Heroin concentration levels in a case A after conditioning in an accustomed place (A1) and in a new place (A2), and in a case B without conditioning.

In this case it could be determined that the heroin used by the patients did not differ in composition from what they had been using earlier. A number of people bought the substance from the same dealer at the same time and subsequently reported that it had not caused them any problem. The concentration of morphine found in the blood was below the morphine values given in the literature in fatal cases; median level: 0.35 mg/l (range: 0.08–3.2 mg/l) [9,10]. This corresponds to the lower limit of morphine levels measured in current heroin users [9]. Probably the user died because he did not take the drug in the accustomed place and circumstances. In the strange, unaccustomed environment the conditioned tolerance described above reducing the effect of the drug action did not operate and a relative overdose resulted (Figure 1). The chance of possible contamination of the heroin powder by actual poisoning substances or infective agents is minimal, since none of those who bought heroin together with the patient had any toxic complications.

In his in-depth study of 99 fatal cases Ingold lists among the risk situations injection of drugs in public places where there was no way of testing the drugs beforehand [11]. This is confirmed by other research [7]. Australian authors have reached the same conclusion: deaths attributed to overdose are likely to have morphine levels no higher than those who survive, or heroin users who die from other causes [8].

The phenomenon of conditioned overdose death is of great significance for harm reduction. Users familiar with the concept of conditioned place preference could have greater chances of survival than those who are not aware of it. This is why there is a need for educational programmes as part of the treatment, making users receiving treatment aware of the nature and risks of conditioning. The more users are aware of the role played by conditioned cues in drug action and in relapse, the greater the chance that they will avoid fatal complications.

We doctors have a great responsibility in alerting the patients we treat to the dangers of conditioning.
